# Transportation Committee

**Published:** 1899-11

**Authors:** W. E. Griswold

**Affiliations:** Denver, Col.


W. E. Griswold, *Sec'y*,

423 Mack Block, Denver, Colo.

### Transportation Committee.

Denver, Col , Oct. 30, 1899.

*Editor Dental Register:*

The Transportation Committee of the National Dental Association for the International Dental Congress in Paris next year are perfecting arrangements for tours, and special rates for delegates and their families, and in all probability they will be completed so as to appear in the January issue of the journals.

W. E. Griswold, *Sec'y*,
Denver, Col.

